# Effects of Transcranial Pulse Stimulation (TPS) on Young Adults With Symptom of Depression: A Pilot Randomised Controlled Trial Protocol

**DOI:** 10.3389/fneur.2022.861214

**Published:** 2022-03-25

**Authors:** Teris Cheung, Yuen Shan Ho, Jerry Wing-Fai Yeung, Sau Fong Leung, Kenneth N. K. Fong, Tommy Fong, Georg S. Kranz, Roland Beisteiner, Calvin Pak Wing Cheng

**Affiliations:** ^1^School of Nursing, The Hong Kong Polytechnic University, Kowloon, Hong Kong SAR, China; ^2^Department of Rehabilitation Sciences, The Hong Kong Polytechnic University, Kowloon, Hong Kong SAR, China; ^3^Department of Psychiatry, The University of Hong Kong, Pokfulam, Hong Kong SAR, China; ^4^Department of Psychiatry and Psychotherapy, Medical University of Vienna, Vienna, Austria; ^5^Department of Neurology, Functional Diagnostics and Therapy, Medical University of Vienna, Vienna, Austria

**Keywords:** transcranial pulse stimulation, brain stimulation, efficacy, major depression (MDD), RCT—randomised controlled trial

## Abstract

**Background:**

Since the emergence of the COVID-19 pandemic, there have been lots of published work examining the association between COVID-19 and mental health, particularly, anxiety and depression in the general populations and disease subpopulations globally. Depression is a debilitating disorder affecting individuals' level of bio-psychological-social functioning across different age groups. Since almost all studies were cross-sectional studies, there seems to be a lack of robust, large-scale, and technological-based interventional studies to restore the general public's optimal psychosocial wellbeing amidst the COVID-19 pandemic. Transcranial pulse stimulation (TPS) is a relatively new non-intrusive brain stimulation (NIBS) technology, and only a paucity of studies was conducted related to the TPS treatment on older adults with mild neurocognitive disorders. However, there is by far no study conducted on young adults with major depressive disorder nationwide. This gives us the impetus to execute the first nationwide study evaluating the efficacy of TPS on the treatment of depression among young adults in Hong Kong.

**Methods:**

This study proposes a two-armed single-blinded randomised controlled trial including TPS as an intervention group and a waitlist control group. Both groups will be measured at baseline (T1), immediately after the intervention (T2), and at the 3- month follow-up (T3).

**Recruitment:**

A total of 30 community-dwelling subjects who are aged 18 and above and diagnosed with major depressive disorder (MDD) will be recruited in this study. All subjects will be computer randomised into either the intervention group or the waitlist control group, balanced by gender and age on a 1:1 ratio.

**Intervention:**

All subjects in each group will have to undertake functional MRI (fMRI) before and after six 30-min TPS sessions, which will be completed in 2 weeks' time.

**Outcomes:**

Baseline measurements and post-TPS evaluation of the psychological outcomes (i.e., depression, cognition, anhedonia, and instrumental activities of daily living) will also be conducted on all participants. A 3-month follow-up period will be used

to assess the long-term sustainability of the TPS intervention. For statistical analysis, ANOVA with repeated measures will be used to analyse data. Missing data were managed by multiple mutations. The level of significance will be set to *p* < 0.05.

**Significance of the Study:**

Results of this study will be used to inform health policy to determine whether TPS could be considered as a top treatment option for MDD.

**Clinical Trial Registration:**

ClinicalTrials.gov, identifier: NCT05006365.

## Introduction

### Background and Rationale

Since the emergence of the COVID-19 pandemic, there have been lots of published work examining the association between COVID-19 and depression in the global general populations (1–3) and vulnerable subpopulations [e.g., frontline nurses (4), students (5), older adults (6), pregnant and postpartum women (7), older adults with psychiatric disorders (8), and migrants (9)]. Major depressive disorder (MDD) is a debilitating disorder affecting individuals' level of bio-psychological-social functioning across different age groups (1). Among all psychiatric disorders, MDD is closely linked to an elevated suicidal risk of ~15–17% (10). Suicide is a complex phenomenon that can be attributed to the following factors: pathophysiological factors, genetic predisposition, exogenous and endogenous stressors, the hypothalamic-pituitary-adrenal stress-response system, the involvement of the monoaminergic neurotransmitter systems, and the deprivation of brain-derived neurotrophic factor and other neuromodulators. Some researchers also investigated the association between affective temperaments and the lifetime suicide-attempt rates on 956 psychiatric in-patients. Results showed that 50% of the MDD participants had lifetime suicide attempts (11). Causations of MDD are multifaceted, and thus, COVID-19 should not be considered as the only aggravator/predictor of MDD without considering other aforementioned factors.

#### Pilot Work Conducted by the Project Team

Our project team has conducted a multi-country cross-sectional study on 10 countries (*N* = 25,053) between March 24 and April 30, 2020 amidst the COVID-19 pandemic (1). Results showed that the young adults aged between 18 and 24 were at higher risks (OR = 5.98 95% CI 3.56, 10.03) of harbouring suicidal ideation (SI) than those older adults aged 60 years or above. Young age was a significant factor associated with SI (1). Notably, the prevalence of depression among Hong Kong citizens was 45.7% (*n* = 5,254), among whom 22% exhibited SI. In addition, Hong Kong had the highest depression prevalence among five Asian collaborative countries in this multi-country study. Prolonged social isolation, loneliness, and social distancing may inevitably increase the likelihood of developing depression during the pandemic and beyond.

#### Local Cross-Sectional Findings Amidst COVID-19 Pandemic

Our project team conducted another cross-sectional study examining the association between depression, face mask use, and health belief in the HK general population between March 24 and April 20, 2020. The overall point-prevalence of probable depression was 46.5% (*n* = 11,072; as defined by Patient Health Questionnaire-9 (PHQ-9) score ≥ 10) (12) during the COVID-19 pandemic. This prevalence estimate was four times higher than the estimate in Hong Kong in late 2019 (11.2%) using the same cut-off score (13).

Our local and international findings were consistent with other researchers (14) who found that 40.4% of the sampled youth between 14 and 35 years old (*n* = 584) reported psychological problems, and 14.4% reported posttraumatic stress symptoms. These pieces of evidence suggest that COVID-19 has a significant impact on youth mental health. Nonetheless, it is not uncommon for young adults with probable depression to avoid seeking professional help. Common reasons for refusal include lack of trust in the treatment and fear of social consequences of help-seeking (15). The personal stigma associated with depression is also a barrier to seeking professional help (16).

Depression is a debilitating disorder affecting individuals' level of bio-psychological-social functioning across different age groups (17). Without timely mental health research (18) and early psychosocial intervention, Hong Kong may soon develop a depression epidemic which may, in turn, increase the global disease burden and healthcare expenditure. Thus, there is a pressing need to formulate effective interventions to mitigate the negative detrimental impact brought by the COVID-19 pandemic (18).

Regarding the clinical manifestation, anhedonia is a core symptom of depression (19) in adolescents (20) and adults (21). The core features of depression are cognitive abnormalities that involve attention, memory, executive functions, and psychomotor speed (22). Impaired cognition, emotional processing, and psychosocial dysfunction are the primary causes of psychosocial dysfunction in depression (23). Nonetheless, research has proven that improvement of anhedonia is positively correlated with improvement in psychosocial functioning among depressive individuals (24).

### Non-invasive Treatment in Psychiatry

Traditionally, pharmacological treatment and psychotherapy have been used as conventional treatment in Psychiatry. However, there have been some other treatment approaches using non-intrusive brain stimulation (NIBS) such as transcranial direct current stimulation (tDCS) (25) and transcranial magnetic stimulation (TMS) (26, 27) in the treatment of depression. The recent development of a new NIBS called “transcranial pulse stimulation” (TPS) has been proven effective for only a 2-week treatment of 35 patients with Alzheimer's disease (AD). Patients' memories had shown improvement which lasted up to 3 months (28). However, there is a paucity of scientific evidence on the efficacy of this TPS intervention on other vulnerable psychiatric populations such as MDD, which is increasingly prevalent in Hong Kong and nationwide, especially during the COVID-19 pandemic (1, 2, 12, 29).

#### How Does TPS Work on the Human Brain?

Transcranial pulse stimulation uses repetitive single ultrashort pulses to stimulate the human brain. With a neuro-navigation device, TPS can target the human brain in a highly focal and precise manner (30). TPS differs from tDCS and TMS as the latter two used direct or induced electric current. Using electric current to stimulate the brain may be limited by the problem of conductivity (31) and failure to reach deep brain regions (32). However, TPS uses low-intensity focused ultrasound which could provide good spatial precision and resolution to modulate subcortical areas, despite the problem of skull attenuation (33). By using lower ultrasound frequencies, TPS can successfully improve skull penetration in the human brain (30).

#### Biological Mechanism of TPS

The basic mechanism of TPS is mechanotransduction. TPS can promote the formation of new blood vessels (angiogenesis) and nerve regeneration, stimulate vascular growth factors (VEGF) (34, 35) and brain-derived neurotrophic factor (BDNF) (36), and improve cerebral blood flow. Mechanotransduction is a biological pathway through which the cells convert the mechanical TPS stimulus into biochemical responses, thus triggering some fundamental cell functions, such as migration, proliferation, differentiation, and apoptosis (37, 38). TPS can stimulate deep cerebral regions (i.e., 8 cm) into the brain. The ultrashort ultrasound pulse could enhance the cell proliferation and differentiation in cultured neural stem cells, which plays an important role in the repair of brain function in central nervous system diseases (39). TPS may affect neurons and induce neuroplastic effects resulting in an increase in cell permeability (39), stimulation of mechanosensitive ion channels (37), and the release of nitric oxide which leads to vasodilation, increased metabolic activity, and angiogenesis (40). Reduced serum levels of BDNF and neuroplasticity were associated with MDD (41, 42).

Since TPS is the latest NIBS device, the underlying mechanism of TPS on how ultrashort ultrasound shock waves may affect neurons and generate neuroplastic effects remain elusive, despite recent TPS studies providing further insight (30, 43). Nonetheless, there are some principles related to different ultrasound-based techniques that could be taken for reference (44). Conducted studies had postulated that the mechanical effects on cell membranes affect mechanosensitive ion channels and generate membrane pores (45) leading to a subsequent change in neurotransmitters and humoral factor concentrations.

Dopamine (DA) is a neurotransmitter that plays an important role in learning, reward, motor control, emotion, and executive functions. Dysfunction of the frontal-striatal dopamine circuit is thought to be involved in depression (46). Thus, frontal-striatal connectivity is a target to manipulate DA activity in deeper brain regions and improve functions that rely on this circuit (47). Past studies used high-frequency repetitive TMS (rTMS) to stimulate the dorsolateral prefrontal cortex (DLPFC), which induced DA release in the ipsilateral caudate nucleus (48) or putamen (49). The DLPFC has dense connectivity with the anterior cingulate cortex (ACC) and the orbitofrontal cortex (OFC). Following high-frequency rTMS of the left DLPFC, there was an increase in DA levels in the ipsilateral ACC and OFC, but no changes occurred in the right DLPFC (50). These findings suggest that DA activity in the human frontal cortex can be manipulated by NIBS. Thus, in this study, we used TPS to probe the frontal-striatal network by targeting the left DLPFC. Apart from DA, it is evident that other neurotransmitters, such as excitatory and inhibitory neurotransmitters glutamate (Glu) and gamma-aminobutyric acid (GABA), are involved in the pathophysiology of MDD (51). Reduction in cortical Glu or GABA levels is associated with MDD. Studies have shown that reduced cortical GABA level is associated with dysfunctional GABAergic interneurons and GABA_A_ receptors, wherein affected GABAergic transmission is believed as a mechanism of MDD (52).

#### Clinical Effects of TPS

Focused ultrasound demonstrated the neuromodulation effect in the human brain. Focused ultrasound can modulate the amplitude of somatosensory evoked potentials (SEPs) when targeted at the cortical regions (53) and the thalamus (33). Conventional treatment approach towards depression in psychiatry includes prescription of rTMS (54), electroconvulsive therapy (55), and anti-depressants, such as selective serotonin reuptake inhibitors (SSRIs), tricyclic antidepressants (TCAs), and serotonin-norepinephrine reuptake inhibitors (SNRIs) (56). All these approaches could increase neuroplasticity and lead to the release of BDNF. Nonetheless, biological treatments alone may not be sufficient to treat all patients with depression, especially if some individuals do not have the normal ability to adapt through neuroplasticity. Thus, psychotherapy is another common treatment approach alongside pharmacology so as to induce clinical improvement of depressive symptoms (57). Nonetheless, ultrasound for the brain is a revolutionary therapeutic treatment approach in patients with neuropsychiatric symptoms. TPS is a novel pulsed ultrasound technique that increases the chances of symptom improvements and may represent an add-on treatment that can be considered with other existing treatment approaches in psychiatry (58).

#### Research Work by Others on TPS

Past research showed that TPS was effective in treating patients with unresponsive wakefulness syndrome. Five patients received 4-week (3 times per week) TPS treatment, having 4,000 pulses each, every 6 months for an average of 2–4 years. Patients showed significant improvement in vigilance, of which three patients' percutaneous endoscopic gastrostomy (PEG) tube could be removed due to improved oropharyngeal motor function (59).

### Previous Study by the Research Team

In another recent study, 35 older adults with AD were treated in 3 TPS sessions, with 6,000 pulses in each session for 2–4 weeks, over the dorsolateral prefrontal cortex, areas of the memory and language network, and/or global brain stimulation. Results showed significant improvement in the Consortium to Establish a Registry for Alzheimer's Disease (CERAD) score (immediately after intervention and at 1- and 3-month after intervention). Functional MRI (fMRI) also showed significantly increased connectivity within the memory network (28). Beisteiner's study also found that participants' depressive symptoms were significantly improved as measured by Geriatric Depression Scale (GDS) (*p* = 0.005) and Beck Depression Inventory (BDI) (*p* < 0.0001) at 1- and 3-months post-stimulation follow-up compared with the baseline scores (28). Increased connectivity in memory network was also evident in Beisteiner's study (60) in the 3-months post-stimulation follow-up. Hence, we believed that a 3-month follow-up time span is sufficient to measure the long-term effects of TPS.

The aims of this study are to (1) evaluate the effects of TPS on symptoms of depression among young adults in Hong Kong; (2) improve young adults' anhedonia symptoms after the 2-week TPS intervention and will be maintained at 3-month post-intervention follow-up; (3) improve young adults' instrumental activities of daily living (IADL) after the 2-week TPS intervention and will be maintained at 3-month post-intervention follow-up; (4) improve young adults' cognition after the 2-week TPS intervention and will be maintained at 3-month post-intervention follow-up; (5) evaluate if there are any structural and functional connectivity in participants' brain after the 2-week TPS intervention compared to pre-TPS MRI scan.

### Objectives

1. **Primary objective**: To evaluate the effects of TPS on symptoms of depression among young adults in Hong Kong.

**Secondary objectives**:

1. To improve young adults' anhedonia symptoms after the 2-week TPS intervention and will be maintained at 3-month post-intervention follow-up
2. To improve young adults' instrumental activities of daily living (IADL) after the 2-week TPS intervention and will be maintained at 3-month post-intervention follow-up.
3. To improve young adults' cognition after the 2-week TPS intervention and will be maintained at 3-month post-intervention follow-up.
4. Post-MRI scan will show a remarkable structural and functional connectivity in participants' brains after the 2-week TPS intervention compared to pre-MRI scan.

### Trial Design

In this proposed study, we will use a single-blind randomised controlled trial design with two-armed repeated measures. The trial design complies with the Consolidated Standards of Reporting Trials (CONSORT) statement (61). In our two-armed design, we will use TPS as an intervention group and a waitlist control group. At the same timepoints, we will appropriately compare the effect of the TPS treatment between those who received it in the intervention group and those who did not receive it in the waitlist control group (62). Both groups were measured at baseline (T_1_), immediately after the intervention (T_2_), and at the 3-month follow-up (T_3_). A total of 30 community-dwelling subjects, aged 18 and above, will be recruited. All subjects will be computer randomised into either the intervention group or the waitlist control group, balanced by gender and age on a 1:1 ratio. All subjects in each group will have to undertake fMRI before and after six 30-min TPS sessions, which will be completed in 2 weeks' time. Baseline measurements and post-TPS evaluation of the psychological outcomes (i.e., depression, cognition, anhedonia, and IADL) will also be conducted on all participants. Based on the previous studies, a 3-month follow-up is sufficient to assess the long-term sustainability of the TPS intervention (60).

## Methods: Participants, Interventions, and Outcomes

### Study Setting

#### TPS Treatment Setting and Treatment Procedures

**Figure 1:**
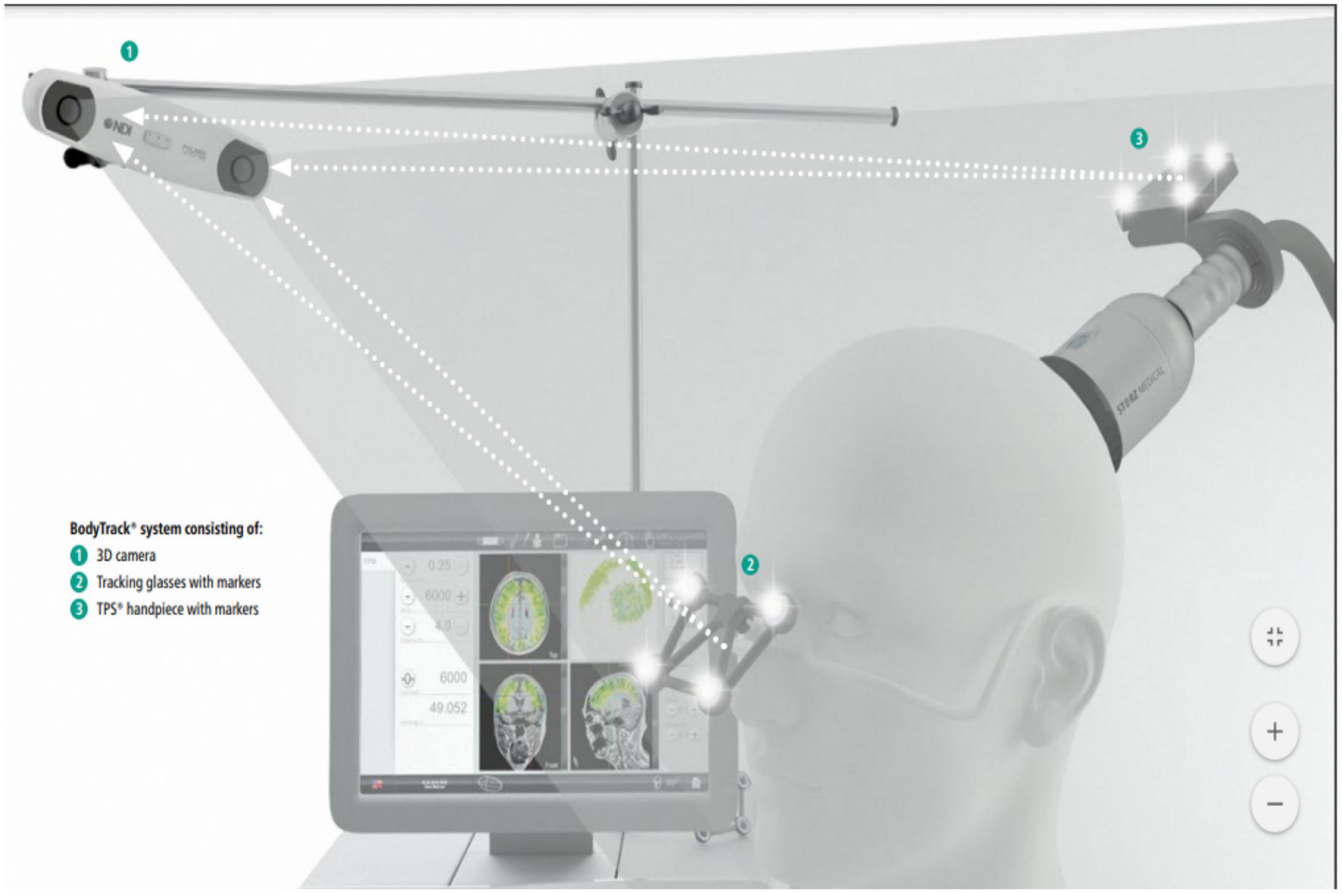
Courtesy image from NEUROLITH-TPS manufacturer.

**Figure 2:**
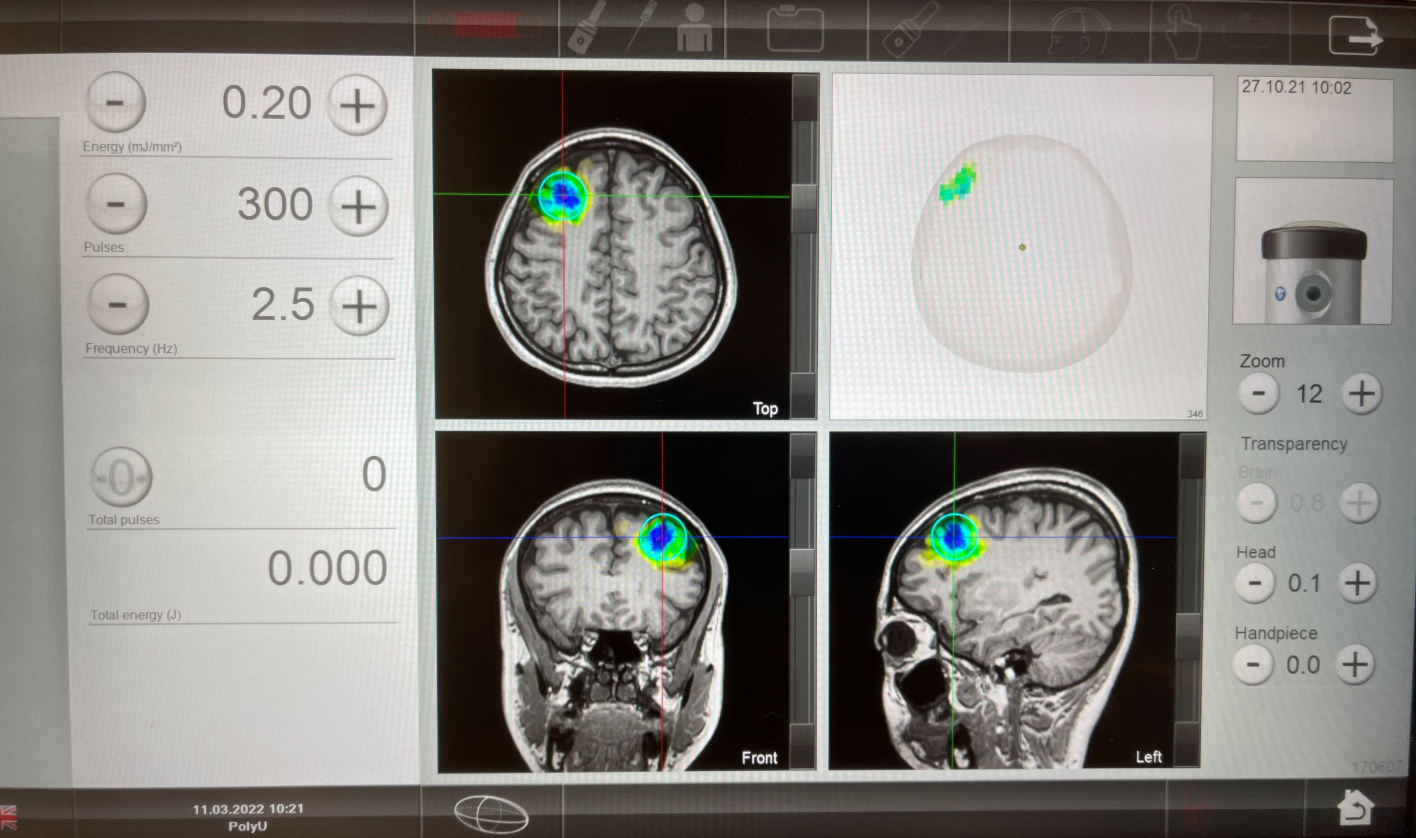
Post TPS stimulation images.

The TPS intervention will be performed at the Integrative Health Clinic, The Hong Kong Polytechnic University. The principal investigator (PI)-licenced medical staff/other trained medical professionals will deliver the intervention. The TPS system consists of a mobile single transducer and an infrared camera system for MR-based neuro-navigation (NEUROLITH, Storz Medical AG, Tägerwilen, Switzerland). TPS generated single ultrashort (3 μs) ultrasound pulses with typical energy levels of 0.2–0.25 mJ/mm^2^ and pulse frequencies of 4–5 Hz (pulses per second). During the TPS session, participants will sit in a comfortable chair in the treatment venue. Participants will wear a BodyTrack system consisting of a 3D camera, tracking glasses with markers, and a TPS handpiece with markers (Figure 1). This BodyTrack system (STORZ MEDICAL AG) will ensure that the participants' heads match with their MRI data so that each applied pulse can be visualised and documented in real-time. Real-time tracking of the handpiece position enabled automatic visualisation of the treated brain region. The energy applied will be highlighted in green colour as shown in Figure 2.

We will target the left DLPFC while guided by real-time MRI brain imaging. The selected brain region is based on previous research (63) in depression that there is an imbalance between left and right DLPFC in patients with MDD. Patients with MDD are associated with hypoactivity in the left DLPFC leading to negative emotional judgment. Past research has proven that stimulation of left DLPFC can effectively improve the depressed mood with different non-invasive brain stimulation techniques such as TMS and tDCS (64). In this study, we will deliver 300 pulses (with a total of 1,800 pulses per subject in 6 sessions, which will be confirmed with NEUROLITH- TPS manufacturer, neurologist, and psychiatrist in the project team) per session to each subject's left DLPFC. The TPS procedure takes 30 min to complete in each session.

**Figure 3:**
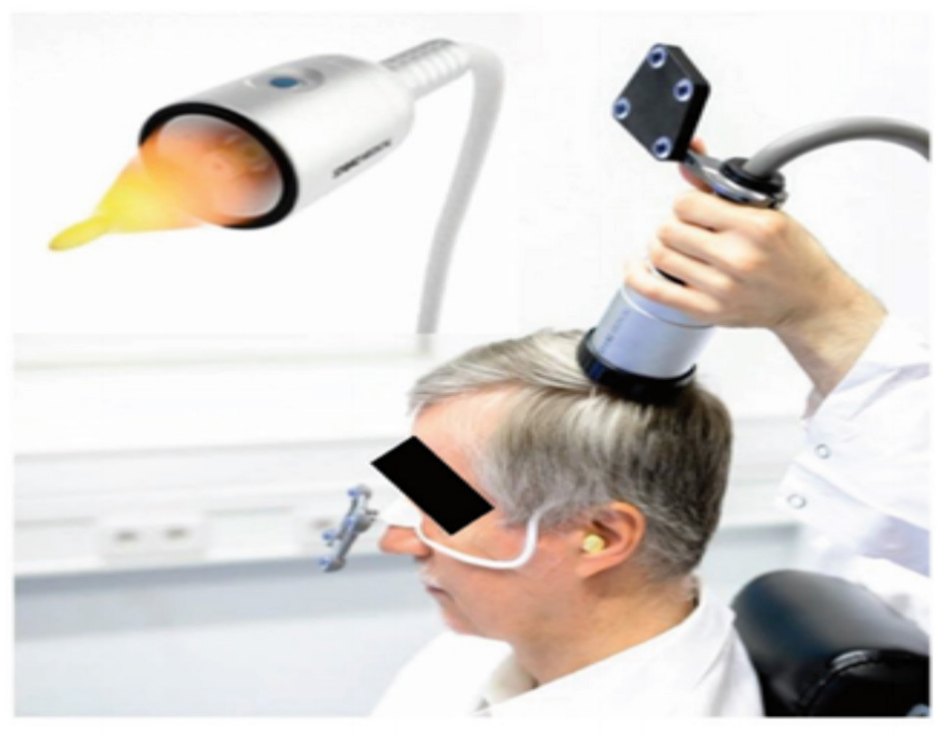
Patient is having a TPS session.

**Figure 4:**
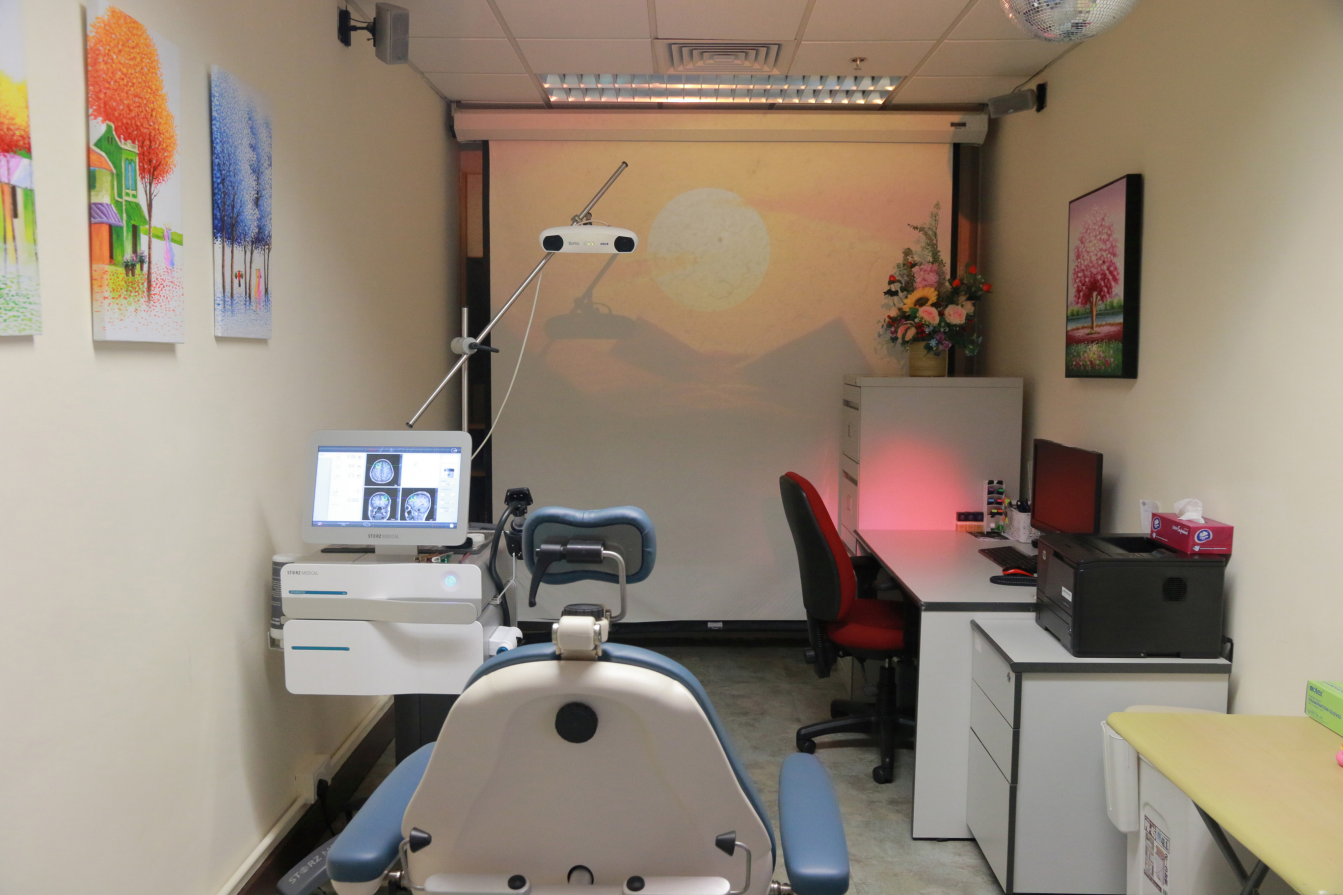
Transcranial Pulse Stimulation System, and TPS treatment venue, The Hong Kong Polytechnic University.

TPS is done *via* variable stand-offs at the handpiece for depth regulation and manual movement of the handpiece over the skull with real-time visualisation of the individual pulses on the patients' MR brain images. The treatment will be done by a trained staff holding the applicator in their hand. The whole treatment session will be recorded for *post-hoc* evaluation of the individual intracerebral pulse localisation (Figure 3). TPS machine is shown in Figure 4.

### Eligibility Criteria

All participants must provide written informed consent before any study procedures occur.

#### Inclusion Criteria

Patients eligible for the trial must comply with all of the following criteria at randomisation: (1) age ≥ 18 years; (2) able to understand/read Chinese; (3) obtain a Hamilton Depression Rating Scale (HAM-D-17) score of ≥ 8.

#### Exclusion Criteria

Patients will be excluded if they corresponded to the following characteristics: (1) individuals being prescribed a DSM-5 diagnosis other than a major depressive disorder (e.g., bipolar affective disorder or schizophrenia); (2) alcohol or substance dependence; (3) concomitant unstable major medical conditions or major neurological conditions such as brain tumour and brain aneurysm; (4) haemophilia or other blood clotting disorders or thrombosis; (5) significant communicative impairments; (6) having a metal implant in the brain or treated area of the head; (7) those who undertook corticosteroid treatment within the last 6 weeks before the first TPS treatment; (8) pregnant or breastfeeding women.

### Who Will Take Informed Consent?

The primary investigator will obtain written informed consent from all subjects in this trial.

### Additional Consent Provisions for Collection and Use of Participant Data and Biological Specimens

The original written consent will cover the approval of receiving neuroimaging (MRI) scan. No biological specimens or ancillary studies will be involved for additional consents.

### Interventions

#### Explanation for the Choice of Comparators

All participants involved will receive TPS treatment. The comparing groups were referred to as the waitlist control group. At the same timepoints, we will appropriately compare the effect of the TPS treatment between those who received it in the intervention group and those who did not receive it in the waitlist control group (62).

#### Intervention Description

##### Intervention (TPS)

**Figure 5:**
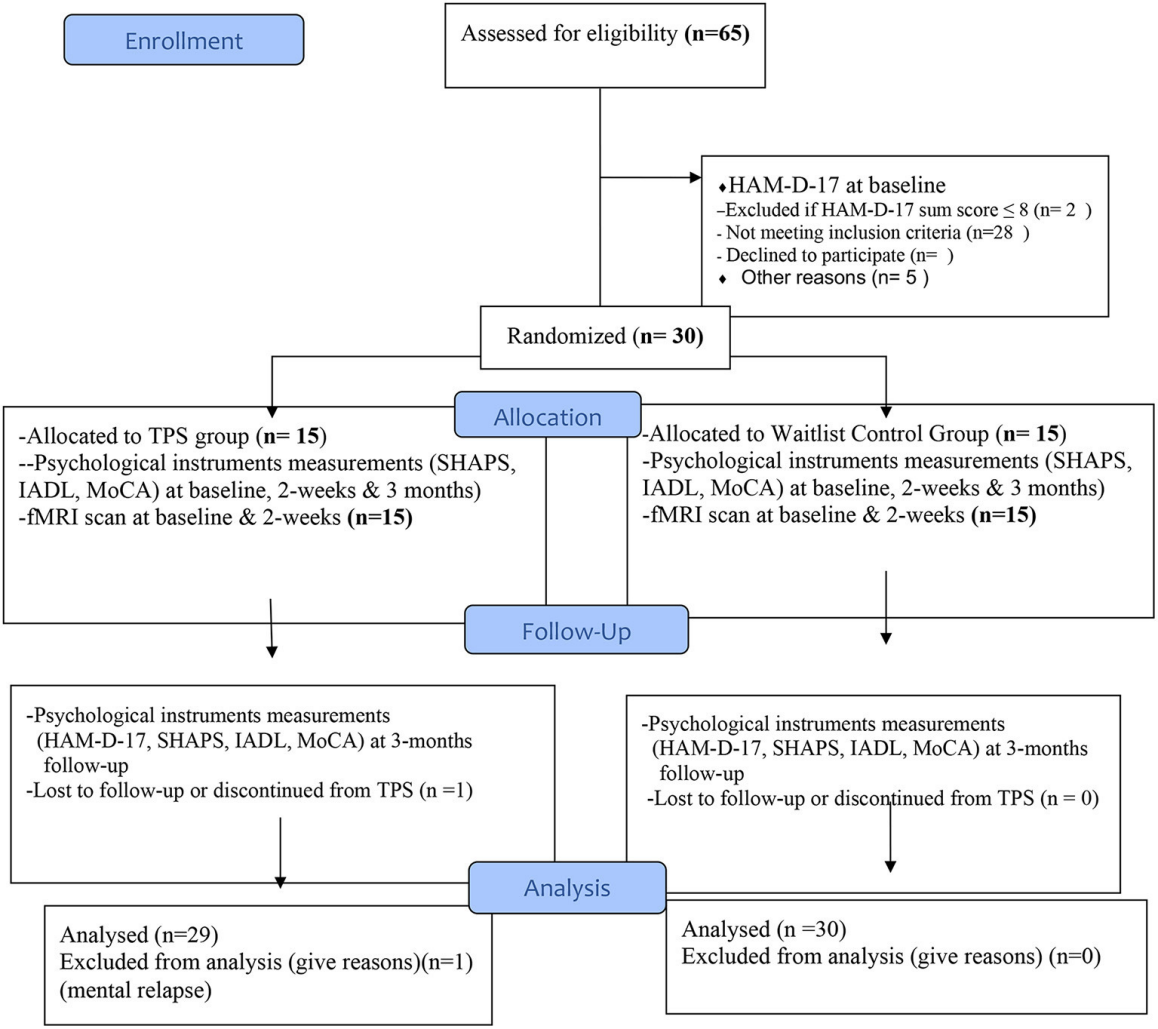
Flow diagram for participants' enrollment, randomisation, allocation, and follow-up (CONSORT, 2010).

**Purpose of the intervention**: The key tenets of the TPS intervention is neuromodulation, i.e., using ultrasound-based brain stimulation techniques to modulate the human brain in a focal and targeted manner (60). **Intervention dose**: Each participant should have the pre-treatment MRI scan performed in the University Research Facility in Behavioural and Systems Neuroscience, PolyU prior to coming to the first intervention session. All participants (both TPS group and the waitlist control group) will receive six 30-min TPS sessions (300 pulses in each session, with a total of 1,800 pulse) in 2 weeks' time (i.e., 3 sessions (Monday, Wednesday, and Friday) per week, a total of 3 h). Participants will be followed up at a 3-month period after the intervention (Figure 5). We believe that a 2-week TPS intervention will be sufficient enough to test the effects of TPS on depressive symptoms (60).

### Criteria for Discontinuing or Modifying Allocated Interventions

The study intervention will be discontinued by trial investigators if any participants have deteriorated health status, exhibited any unexpected adverse effects towards the TPS intervention, or withdrawal of participant consent. The trial investigators will either modify or discontinue the intervention. Nonetheless, study participants will be retained in the trial to enable follow-up data collection and prevent missing data.

### Strategies to Improve Adherence to Interventions

**Fidelity of the intervention:** To ensure the fidelity of the intervention, the project team will ascertain whether the interventions are delivered as designed. A WhatsApp message reminder (e.g., TPS intervention schedule, fMRI scan appointments, and f/u appointments) will be delivered to participants to monitor their progress and adherence throughout the TPS study.

### Interventions—Concomitant Care

Participants will be instructed continue to take their prescribed medications for other conditions as normal. Self-prescription of any psychotropic medications to mitigate depressive symptoms is strongly discouraged to avoid contamination of data. Throughout the intervention period, participants will be advised to avoid strenuous exercise and adhere to healthy lifestyles and regular resting periods.

### Provisions for Post-trial Care

Participants who are finished receiving the TPS treatment will have a follow-up assessment at a 3-month period. They could contact the PI during the post-trial for any potential physical or mental health issues related to the TPS treatment.

## Outcomes

### Outcome Measures (Primary and Secondary Outcomes)

#### Baseline Assessments

##### Diagnostic Interview

The diagnosis of MDD will be confirmed using the Structured Clinical Interview for Diagnostic and Statistical Manual of Mental Disorders (SCID-5).

##### Demographic Data

We will solicit the demographic data of the patients, including age, gender, educational attainment, place of birth, marital status, number of children, financial condition, family history of depression/mood disorders, and family household income. Medical comorbidities will be assessed with the Cumulative Illness Rating Scale (CIRS). The handedness of the participants will be evaluated using the Edinburgh Handedness Inventory—Short Form. Participants' medical and psychiatric history, including the age of onset of major physical illnesses, depressive symptoms (with or without current medication), and treatment /drug dosage, will also be recorded at baseline.

#### Primary Outcomes

##### Depression

Hamilton Depression Rating Scale-17 (65) will be used to measure symptoms of depression and participants' mood and depression severity. HAM-D-17 is a widely used reliable measurement of depressive and mood symptoms. Scores will range from 0 to 52, with higher scores indicating more severe depression. Clinical response will be defined as a reduction of 50% or more in the HAM-D-17 score. A HAM-D-17 score of 7 or less will be used as an indicator of remission.

#### Secondary Outcomes

##### Anhedonia

The core symptom of depression will be assessed by the Chinese version of the Snaith-Hamilton Pleasure Scale (SHAPS; clinical utility of the SHAPS in Chinese settings). SHAPS is a 14-item scale assessing participants' hedonic experience in the last few days. SHAPS includes four domains (interest/pastimes, social interaction, sensory experience, and food/drink), rated on a 4-point Likert-type scale (1, Definitely Agree; 2, Agree; 3, Disagree; 4, Definitely Disagree). Total score will range from 14 to 56, with higher scores representing more pronounced anhedonia. It is one of the most widely used self-report questionnaires in clinical research for evaluating anhedonia with good psychometric properties (66).

#### Instrumental Activities of Daily Living (IADL)

Instrumental activities of daily living will be assessed by the Hong Kong Chinese version of the Lawton Instrumental Activities of Daily Living Scale. This IADL is a valid and reliable tool to assess the daily functioning of Hong Kong adults (67). The scale encompasses nine activities which include the ability to use the telephone, shopping, food preparation, housekeeping, laundry, home maintenance, transport, ability to handle finances, and responsibility for own medication. Participants will be instructed to indicate their ability on a 4-point Likert scale (0, Definitely not; 1, Need some help; 2, Can do it by myself but with some difficulty; 3, Do not need any help). The total score of the IADL will range from 0 to 27, with higher scores indicating more independence in activities of daily living.

#### Cognition

Global cognition will be measured using the Hong Kong Chinese version of the Montreal Cognitive Assessment (MoCA) (68). The MoCA is a one-page test with a maximum score of 30. One point is added if the person has 12 years of education or less. A score of 23–26 represents mild cognitive impairment, 17–22 represents moderate impairment, and 16 or below suggests severe impairment and dementia. Working memory, executive function, and attention will be measured by forward and backward digit span, the category verbal fluency test, and the Trail Making Test (TMT) Parts A and B.

#### Neuroimaging

Participants will receive pre- and post-treatment MRI scans to measure any changes in structural and functional connectivity changes in the brain. Structural MRI, diffusion tensor imaging (DTI), and resting state-fMRI (rs-fMRI) will be performed using a 3T scanner at the University Research Facility in Behavioural and Systems Neuroscience, The Hong Kong Polytechnic University. The subjects will be closely monitored by the research assistant and the radiographer during scanning. The whole scan will last around 45 min including preparation. Structural MRI scans, including T1- and T2-weighted fluid attenuation inversion recovery (T2-FLAIR) sequences, will be used for assessing regional volume differences across the whole brain. High resolution sagittal 3D T1-weighted [spoiled gradient recalled echo (SPGR)/magnetisation-prepared rapid gradient echo (MPRAGE)] images of 1 mm x 1 mm x 1 mm were acquired with repetition time (TR) = 1,820 ms, echo time (TE) = 3.75 ms, inversion time (TI) = 1,100 ms, and flip angle = 70°. This will be followed with axial 2D-T2-weighted FLAIR images of 0.9 × 0.9 × 3 mm, which will be acquired with TR = 9,160 ms, TE = 90 ms, TI = 2,500 ms, and flip angle = 150°, for the quantification of white matter hyperintensities (WMH). DTI sequence will be conducted using a single-shot spin-echo echo-planar imaging, with diffusion-sensitising gradients applied along 16 non-collinear directions with diffusion weighting factor b = 1,000 s/mm^2^ and two b = 0 images. The imaging parameters were the following: TR/TE = 1,200/82 ms, matrix size = 128 × 128, field of view (FOV) = 240 mm, slice thickness = 3 mm with no intersection gap, number of excitations = 2, and number of slices = 67. Finally, resting-state fMRI of 150 T2-weighted gradient echo-planar imaging (EPI) will be acquired with TR = 2 s and TE = 32 ms, with 32 slices, and a resolution of 3 × 3 × 4 mm, during which subjects will view a fixation cross (“+”) passively at the centre of the screen. Images processing and analysis will be performed using software packages including FSL (http://fsl.fmrib.ox.ac.uk/fsl/fslwiki/), Freesurfer, and SPM8 (Oxford, UK). Total brain and its total grey and white matter volumes will be extracted from the T1 structural scan. Grey matter and white matter tissue maps will be segmented and compared for regional tissue density differences using voxel-based morphometry (VBM) (69). Structural connectivity will be assessed by fractional anisotropy (FA) maps extracted from DTI imaging.

For functional connectivity, all resting state-fMRI (rs-fMRI) volumes will be pre-processed, with motion correction and slice timing correction, and then linearly registered to the Montreal Neurological Institute (MNI) standard space. A data-driven approach will be used for the analysis of rs-fMRI data. Independent component analysis will be done with Multivariate Exploratory Linear Decomposition in FSL. A set of independent components will be identified as the common resting-state functional networks. The global and local efficiency, modularity, and hubs will be computed using the Brain Connectivity Toolbox (https://sites.google.com/site/bctnet/). A dual regression approach will be used to investigate between-group differences in the individual functional networks. The significance threshold of the voxel-wise differences will be set at *p* < 0.05 (family-wise error corrected).

### Participant Timeline

Each participant will have to undertake pre-and post fMRI and resting-MRI scan in this trial. They will undertake a 2-week TPS treatment, with three 30-min sessions each week. It takes ~1 month to complete all the interventions, and all participants will be followed-up at a 3-month period after the last TPS treatment session.

### Sample Size

#### Sample Size Estimation

To the best of our knowledge, there is only one TPS uncontrolled pilot study conducted on 35 patients with AD in Austria (60). Therefore, we cannot base on their effect size to estimate our sample size in this study. Considering the nature of our study is the first pilot RCT in using TPS in the treatment of depression, and also referenced by Beisteiner's study, we aim to recruiting 30 subjects to evaluate the efficacy of our primary and secondary outcomes in this study.

### Recruitment

Participants will be recruited *via* a mass email invitation delivered to the eligible subjects *via* collaborators in The Hong Kong Polytechnic University and University of Hong Kong. A QR code flyer will also be flagged up in communal areas in The Hong Kong Polytechnic University and University of Hong Kong campuses. A 1-h orientation session on TPS intervention will be conducted at The Hong Kong Polytechnic University and University of Hong Kong to explain the purpose and objectives of the study and to recruit potential participants on site. The research assistant will be stationed on-site in The Hong Kong Polytechnic University and University of Hong Kong to facilitate recruitment and answer immediate enquiries.

## Assignment of Interventions: Allocation

### Sequence Generation

#### Randomisation, Allocation, and Masking

All consenting participants will be listed in alphabetical order according to their surnames, and each participant will be assigned a unique study number. After baseline measurement, randomisation will be conducted by an independent statistician off-site using a stochastic minimisation programme to balance the gender, age, and depression scores of the participants. Eligible participants will be randomised into two groups using a pre-/post-test waitlist control group design. The allocation to the two groups will be 1:1.

### Concealment Mechanism

An independent statistician will use a computer-generated list of random numbers (www.random.org) to ensure concealment of randomisation. To avoid information flow, participants in both groups will be advised not to reveal their study participants to the other group, to minimise potential contamination of the effects of TPS or subject bias. The participant list will be concealed from the recruitment venues throughout the study.

### Implementation

The PI and research assistants will enrol participants, and the PI will assign participants to either the TPS group or the waitlist control group.

## Assignment of Interventions: Blinding

### Who Will Be Blinded

Trial participants from both intervention and waitlist control groups will be blinded after assignment to groupings before treatment.

### Procedure for Unblinding If Needed

Participants will be asked to guess which group they belonged to in the last TPS session to evaluate the success of blinding.

### Data Collection and Management

#### Plans for Assessment and Collection of Outcomes

Both groups will be measured at baseline (T_1_), immediately after the intervention (T_2_), and at the 3-month follow-up (T_3_; refer to Figure 5 CONSORT flow diagram). Based on the previous studies, a 3-month follow-up is sufficient to assess the long-term sustainability of the TPS intervention (60). A trained clinician investigator who is not involved in the intervention will perform the assessments. The primary and secondary outcomes will be assessed at baseline, immediately after the intervention, and 12 weeks after the intervention.

#### Plans to Promote Participant Retention and Complete Follow-Up

A WhatsApp message reminder (e.g., TPS intervention schedule, fMRI scan appointments, and f/u appointments) will be delivered to participants to monitor their progress and adherence throughout the TPS study.

### Data Management

#### Data Management Plan (DMP)

The DMP is enforced according to the Personal Data (Privacy) Ordinance, Code of Ethics for Research Involving Human Subjects, and the Policy on Ownership of Intellectual Property issued by the PolyU. All the anonymized data including fMRI scans, laboratory results and documentation will be converted into.csv, and.pdf formats for long-term preservation and access and stored in a Figshare and PolyU home drive for 5 years with a DataCite DOI as an identifier.

### Confidentiality

All consenting participants will be listed in alphabetical order according to their surnames, and each participant will be assigned a unique study number. All personal information collected for potential and enrolled participants will only be shared under the supervision of the principal investigator. All confidential data will be kept and maintained by the principal investigator after the trial ends in case any emergencies will be raised from participants. The confidential data will be destroyed once publications have been made.

### Plans for Collection, Laboratory Evaluation, and Storage of Biological Specimens for Genetic or Molecular Analysis in This Trial/Future Use

Not applicable.

### Statistical Methods

#### Statistical Methods for Primary and Secondary Outcomes

##### Data Analysis Plan

Data will be analysed by the SPSS for Windows, version 27. The normality of the primary outcome (depression) scores will be tested by the Kolmogorov-Smirnov test. If significant differences between sociodemographic variables are found, covariates will be used as a control or confounding variables in the main analysis. For Objective 1, multilevel modelling analysis will be used to investigate changes in the outcome measures (depression) at each time interval (2 weeks and 3 months) with respect to their baseline measurements to examine the effects of TPS. A two-level model will be built, with repeated time points at level 1 and respondents at level 2. The baseline difference between groups will be tested using the Wald test based on the regression coefficient estimates of the indicator. The changes in the outcome measures over time will be included in the fixed part of the model to estimate change from baseline to 2 weeks and from baseline to 3 months. To assess the effects of TPS over time, interaction terms will be included in the fixed part of the model. The significantly different demographics between groups identified in step one of the analysis and other possible confounders will be adjusted by including them in the main-effect model. The within-respondent random effects of the outcome measurements will be taken into account by the level-2 variance in the model. For Objectives 2, 3, and 4, pairwise comparison and other hypotheses on the intervention effects will be tested using a generalised Wald test with an adequate degree of freedom from the modelling analysis (70). The same model, which will be conducted separately for each outcome, will be used to investigate the effect of interventions on secondary outcome measures. To assess the effects of TPS, an intention-to-treat (ITT) analysis will be performed on all participants who undergo random allocation. A multiple imputation method will be used to handle missing data. The pooled results of the multilevel analyses with five multiple imputations will be presented. A sensitivity analysis will be performed by comparing results from analysis datasets with and without missing data imputation. In all tests, significant differences between groups will be detected with a 95% CI, and *p*-values of < 0.05 (two-tailed) will be considered significant. For Objective 5, the neurological rs-fMRI data, longitudinal VBM will be used to examine whether TPS produces local changes in grey matter. Specifically, relative local increases and decreases between pre- and post-intervention scans will be compared within and between study groups. The diffusion MR data will be analysed using the diffusion tensor model. After a mathematical diagonalization process, the eigenvectors and eigenvalues describing the tensor ellipsoid will be determined. Then, two standard diffusion indices will be obtained which are the following: (1) the apparent diffusion coefficient and (2) the fractional anisotropy. DTI scans pre- and post-treatment will permit the assessment of changes in axial diffusivity, with lower values being interpreted as the structural enhancement of white matter. For the cognitive data analysis, general linear modelling (GLM) will be used to compare the difference in performance in the forward and backward digit span, the Stroop test, the category verbal fluency test, and the TMT Parts A and B between the TPS group and the waitlist control group. Performance on the TMT between the TPS group and the waitlist control group will be analysed by one-way repeated-measures ANOVA. Participants' narrative feedback on TPS will be analysed with descriptive content analysis.

### Interim Analyses

Only the PI will have access to all participants' data. Should any participants suffer from any adverse effect during or after the TPS treatment, they will be duly assessed by the PI and TPS treatment will be terminated as deemed necessary.

### Methods for Additional Analyses (E.g., Subgroup Analyses)

Subgroup analyses will be conducted between the TPS group and the waitlist control group in terms of the primary and secondary outcomes.

### Methods in Analysis to Handle Protocol Non-adherence and Any Statistical Methods to Handle Missing Data

Multiple imputations will be used to manage missing data in any treatment phrases.

### Plans to Give Access to the Full Protocol, Participant Level-Data, and Statistical Code

Anonymous data is available on request.

## Oversight and Monitoring

### Composition of the Coordinating Centre and Trial Steering Committee

Since this is a small-scale study comprising 30 subjects, the PI will closely monitor the progress and intervention of this trial. In addition, the PI will organise regular group meetings with the project team members to update the progress of the study until the completion of this trial.

### Composition of the Data Monitoring Committee, Its Role, and Reporting Structure

Data monitoring committee is not required in this trial as there is no competing interest between the sponsor and the project team members. The sponsor is not involved in the research design, data collection, and execution of this study.

### Adverse Event Reporting and Harms

#### The Safety Issue of TPS

Transcranial pulse stimulation uses very low energy for brain stimulation. *In vivo* animal TPS study did not cause any tissue damage despite using 6- to 7-fold higher energy levels compared with those in human studies. Furthermore, the intervention did not cause any serious adverse effects such as intracranial bleeding, oedema or other intracranial pathology, as confirmed with MRI in a previous AD study. Few subjects reported headache (4%), pain or pressure (1%), and mood deterioration (3%) (60). The Clinically Certified (CE) marked TPS system has proven to be safe in more than 1,500 treatments.

#### Adverse Effects and Risk Indicators

A checklist of potential adverse effects associated with TPS administration will be generated from existing literature (30). The checklist will be used to monitor tolerability and adverse events in each session throughout the intervention.

### Frequency and Plans for Auditing Trial Conduct

The PI will be responsible to conduct random and non-random audit throughout the trial period, ensuring that the protocol and TPS intervention has strictly adhered to protocol.

### Plans for Communicating Important Protocol Amendments to Relevant Parties (E.g., Trial Participants, Ethical Committees)

Should there be any changes in the eligibility criteria or outcome measurements, IRB and research ethics committee in The Hong Kong Polytechnic University will be immediately notified.

### Dissemination Plans

Investigators will disseminate the study findings *via* international journal publications, international conferences, and press conferences.

## Discussion

Our results will highlight the efficacy and long-term sustainability of TPS in the treatment of depressive symptoms among young adults. Our findings may add new knowledge on the efficacy of using TPS as a non-intrusive, and non-pharmacological treatment of depression in Psychiatry. It will also lay down the groundwork for a larger clinical trial in the near future in order to evaluate if this new treatment modality in psychiatry can be used to treat patients suffering from other types of neuropsychiatric disorders.

## Conclusion

Using NIBS in the treatment of neuropsychiatric disorders is the future trend in the field of psychiatry and neuroscience. Existing findings have already proven that TPS is an effective treatment modality in Alzheimer's Disease. Our study findings will further support the notion that TPS can also be used in the treatment of MDD. Study findings may have a significant impact on researchers/clinicians, individuals who are treatment-resistant to all sorts of antidepressants, and/or those who have poor drug compliance and insight into their neuropsychiatric disorders.

### Research Gap

Since TPS is a relatively new NIBS technology, only a paucity of studies was conducted on older adults with mild NCD (60). There is no study conducted on young adults with depression in Hong Kong and nationwide. This gives us the impetus to propose this study to bridge the research gap and to evaluate the efficacy of TPS on the treatment of depression in young adults in Hong Kong.

### Innovations of the Study

To the best of our knowledge, our proposed study is the first pilot study to use TPS to treat young adults with depression in Europe, Asia, and Hong Kong.

### Research Impact and Significance of the Study

Health policy-makers and researchers are working to ameliorate hard to formulate effective mental health interventions to curb the depression epidemic brought by the pandemic situations (71, 72) and beyond. It is envisaged that our findings can be transferable to other cultural contexts. Data emerging from this study will facilitate cross-cultural and interdisciplinary collaboration to provide an evidence-based treatment for individuals with probable depression. Our findings may add new knowledge on the efficacy of using TPS as a non-intrusive and non-pharmacological treatment of depression. It also lays down the groundwork for a larger clinical trial in the near future, to evaluate if this new treatment modality in psychiatry can be used to treat patients suffering from other types of psychiatric disorders.

### Limitations of This Study

There are some limitations that need to be addressed in this protocol. First, since we will execute the first pilot RCT nationwide interrogating the effects of TPS on MDD, we can only use existing NIBS studies like TMS and tDCS to act as a comparison for our findings. Since both TMS and tDCS use non-invasive neuromodulation technology by electrical stimulation, the mechanism will be somehow different from TPS which uses ultrashort ultrasound waves in the stimulation of the human brain. Thus, our findings will need to be interpreted with caution. Second, participants in the waitlist control group will be given the identical TPS intervention, albeit in different timepoints, but they will all receive the same TPS pulse/frequency/energy in this TPS trial. Third, the TPS machine itself may have a potential placebo effect which may bias the results. Fourth, there could be a potential risk of contamination if our waitlist control group utilised self-prescribed pharmaceutical input or other TCM modalities (e.g., herbal soup and acupuncture) to treat their symptoms of depression while waiting the TPS treatment. Such contamination may impact the receptivity and efficacy of our TPS intervention. Fifth, participants will be administered the same sets of psychological instruments at three different time points so there is a potential risk that some participants may recall some contents of these instruments. To prevent this, our research personnel will not provide accurate answers to these tests throughout each study phase to minimise recall bias. Last but not least, individuals with severe depression with fleeting suicidal ideas will be excluded in this study, and thus, the efficacy of TPS on these severely depressive and suicidal community-dwelling individuals will remain unknown. Future replication of a multi-centre study may adopt a double-blinded, sham-controlled RCT to philtre out the placebo effects on a larger sample.

### Trial Status

This trial was registered with ClinicalTrials.gov on August 16, 2021 (protocol version). Recruitment commenced on 1 August 2021 and was completed on 30 October 2021.

## Data Availability Statement

The original contributions presented in the study are included in the article/supplementary material, further inquiries can be directed to the corresponding author/s.

## Ethics Statement

All participants are required to provide written informed consent to participate in this study. Ethical approval will be sought from the Human Subjects Ethics Sub-committee, The Hong Kong Polytechnic University. Research Safety Approval will also be obtained prior to the commencement of the study. This study adheres strictly to the Declaration of Helsinki ethical principles developed by the World Medical Association. All participants will be covered by trial insurance. Potential risks involved in fMRI will be clearly indicated in the Information Sheet. Voluntary participation, anonymity and confidentiality, and the right to withdraw are respected. All participants will be given a HK$50 supermarket cash coupon for their time and travel-related expenses for the pre-and post-treatment fMRI.

## Author Contributions

TC, YH, KF, RB, CC, and GK conceived the study. TC and CC designed, executed the study, administered the TPS, and arranged neuroimaging logistics. YH assisted in the administration of TPS and baseline and post-TPS measurements. GK, CC, and TF assisted in data entry and administration of baseline and post-TPS measurements. RB, KF, and CC offered expert advice in fMRI data analysis and manuscript writing. All authors contributed to the article and approved the submitted version.

## Funding

This work was supported by the Departmental General Research Funds, The Hong Kong Polytechnic University, Hong Kong (Grant Number P0036773). RB is supported by the Herzfelder Stiftung, Austria.

## Conflict of Interest

The authors declare that the research was conducted in the absence of any commercial or financial relationships that could be construed as a potential conflict of interest.

## Publisher's Note

All claims expressed in this article are solely those of the authors and do not necessarily represent those of their affiliated organizations, or those of the publisher, the editors and the reviewers. Any product that may be evaluated in this article, or claim that may be made by its manufacturer, is not guaranteed or endorsed by the publisher.
